# A Space–Time Permutation Scan Statistic for Disease Outbreak Detection

**DOI:** 10.1371/journal.pmed.0020059

**Published:** 2005-02-15

**Authors:** Martin Kulldorff, Richard Heffernan, Jessica Hartman, Renato Assunção, Farzad Mostashari

**Affiliations:** **1**Department of Ambulatory Care and Prevention, Harvard Medical School and Harvard Pilgrim Health CareBoston, MassachusettsUnited States of America; **2**New York City Department of Health and Mental Hygiene, New YorkNew YorkUnited States of America; **3**New York Academy of Medicine, New YorkNew YorkUnited States of America; **4**Departamento de Estatistica, Universidade Federal de Minas GeraisBelo Horizonte, Minas GeraisBrazil; University of California at Los AngelesUnited States of America

## Abstract

**Background:**

The ability to detect disease outbreaks early is important in order to minimize morbidity and mortality through timely implementation of disease prevention and control measures. Many national, state, and local health departments are launching disease surveillance systems with daily analyses of hospital emergency department visits, ambulance dispatch calls, or pharmacy sales for which population-at-risk information is unavailable or irrelevant.

**Methods and Findings:**

We propose a prospective space–time permutation scan statistic for the early detection of disease outbreaks that uses only case numbers, with no need for population-at-risk data. It makes minimal assumptions about the time, geographical location, or size of the outbreak, and it adjusts for natural purely spatial and purely temporal variation. The new method was evaluated using daily analyses of hospital emergency department visits in New York City. Four of the five strongest signals were likely local precursors to citywide outbreaks due to rotavirus, norovirus, and influenza. The number of false signals was at most modest.

**Conclusion:**

If such results hold up over longer study times and in other locations, the space–time permutation scan statistic will be an important tool for local and national health departments that are setting up early disease detection surveillance systems.

## Abstract 2

A new, flexible method for disease outbreak surveillance and its application to emergency department data from New York City

## Introduction

The World Trade Center and anthrax terrorist attacks in 2001, as well as the recent West Nile virus and SARS outbreaks, have motivated many public health departments to develop early disease outbreak detection systems using non-diagnostic information, often derived from electronic data collected for other purposes (“syndromic surveillance”) [1,2,3,4,5,6,7,8,9,10,11,12,13,14,15,16,17]. These include systems that monitor the number of emergency department visits, primary care visits, ambulance dispatches, nurse hot line calls, pharmaceutical sales, and West Nile–related dead bird reports. The establishment of such systems involves many challenges in data collection, analytical methods, signal interpretation, and response. Important analytical challenges include dealing with the unknown time, place, and size of an outbreak, detecting an outbreak as early as possible, adjusting for natural temporal and geographical variation, and dealing with the lack of suitable population-at-risk data.

Most analytical methods in use for the early detection of disease outbreaks are purely temporal in nature [18,19,20,21,22]. These methods are useful for detecting outbreaks that simultaneously affect all parts of the geographical region under surveillance, but may be late at detecting outbreaks that start locally. While purely temporal methods can be used in parallel for overlapping areas of different sizes in order to cover all possible outbreaks, that approach leads to a severe problem of multiple testing, generating many more false signals than the nominal statistical significance level would indicate.

First studied by Naus [23], the scan statistic is an elegant way to solve problems of multiple testing when there are closely overlapping spatial areas and/or time intervals being evaluated. Temporal, spatial, and space–time scan statistics [24,25,26,27] are now commonly used for disease cluster detection and evaluation, for a wide variety of diseases including cancer [28,29], Creutzfeldt-Jakob disease [30], granulocytic ehrlichiosis [31], sclerosis [32], and diabetes [33]. The basic idea is that there is a scanning window that moves across space and/or time. For each location and size of the window, the number of observed and expected cases is counted. Among these, the most “unusual” excess of observed cases is noted. The statistical significance of this cluster is then evaluated taking into account the multiple testing stemming from the many potential cluster locations and sizes evaluated.

To date, all scan statistics require either a uniform population at risk, a control group, or other data that provide information about the geographical and temporal distribution of the underlying population at risk. Census population numbers are useful as a denominator for cancer, birth defects, and other registry data, where the expected number of cases can be accurately estimated based on the underlying population. They are less relevant for surveillance data such as emergency department visits and pharmacy sales, since the catchment area for each hospital/pharmacy is undefined. Even if it were available, the catchment area population would not be a good denominator since there can be significant natural geographical variation in health-care utilization data, due to disparities in disease prevalence, access to health care, and consumer behavior [34]. One option when evaluating data that are affected by utilization behavior is to use total volume as the denominator. For example, one may use total emergency department visits as a denominator when evaluating diarrhea visits [7], or similarly, all pharmacy sales as the denominator when evaluating diarrhea medication sales [4]. This may or may not work depending on the nature of the data. For example, changes in total drug sales due to sales promotions or the allergy season could hide a true signal or create a false signal for the drug category of interest.

In this paper we present a prospective space–time permutation scan statistic that does not require population-at-risk data, and which can be used for the early detection of disease outbreaks when only the number of cases is available. The method can be used prospectively to regularly scan a geographical region for outbreaks of any location and any size. For each location and size, it looks at potential one-day as well as multi-day outbreaks, in order to quickly detect a rapidly rising outbreak and still have power to detect a slowly emerging outbreak by combining information from multiple days.

The space–time permutation scan statistic was gradually developed as part of the New York City Department of Health and Mental Hygiene (DOHMH) surveillance initiatives, in parallel with the adaptation of population-at-risk-based scan statistics for dead bird reports (for West Nile virus) [13], emergency department visits [7], ambulance dispatch calls [6], pharmacy sales [4], and student absentee records [3]. In this methodological paper, the space–time permutation scan statistic is presented and illustrated using emergency department visits for diarrhea, respiratory, and fever/flu-like illnesses.

## Methods

### New York City Emergency Department Syndromic Surveillance System

The New York City Emergency Department syndromic surveillance system is described in detail elsewhere [7]. In brief, participating hospitals transmit electronic files to the DOHMH seven days per week. Files contain data for all emergency department patient visits on the previous day, including the time of visit, patient age, gender, home zip code, and chief complaint—a free-text field that captures the patient's own description of their illness. As of November 2002, 38 of New York City's 66 emergency departments were participating in the system, covering an estimated 75% of emergency department visits in the city.

Data are verified for completeness and accuracy, concatenated into a single dataset, and appended to a master archive using SAS [35]. To categorize visits into “syndromes” (e.g., “diarrhea syndrome”), a computer algorithm searches the free-text chief complaint field for character strings indicating symptoms consistent with that syndrome.

The goal of data analysis, which is carried out seven days per week, is to detect unusual increases in key syndrome categories. To run the space–time permutation scan statistic we have written a SAS program that generates the necessary case and parameter files, invokes the SaTScan software [36], and reads the results back into SAS for reporting and display.

Two sets of analyses are performed, one based on assigning each individual to the coordinates of their residential zip code and the other based on their hospital address. With 183 zip codes versus 38 hospitals, the former utilizes more detailed geographical information, while the latter may be able to pick up outbreaks not only related to place of residence but also to place of work or other outside activities (if people go to the nearest hospital when they feel sick). Residential zip code is not recorded by the hospital for about 3% of patients, and for the analyses described here, these individuals are only included in the hospital-based analyses.

The performance of the prospective space–time permutation scan statistic was evaluated using both hospital and residential analyses. We used historical diarrhea data to mimic a prospective surveillance system with daily analyses from 15 November 2001 to 14 November 2002. For each of these days, the analysis only used data prior to and including the day in question, ignoring all data from subsequent days. This corresponds to the actual data available at the DOHMH 8–12 h after the end of that day, when that analysis would have been conducted if the system has been in place at that time. We also present one week of daily prospective analyses conducted in November 2003, where the daily analysis was run about 12 h after the conclusion of each day, as part of the regular syndromic surveillance activities at the DOHMH.

### The Space–Time Permutation Scan Statistic

As with the Poisson- and Bernoulli-based prospective space–time scan statistics [27], the space–time permutation scan statistic utilizes thousands or millions of overlapping cylinders to define the scanning window, each being a possible candidate for an outbreak. The circular base represents the geographical area of the potential outbreak. A typical approach is to first iterate over a finite number geographical grid points and then gradually increase the circle radius from zero to some maximum value defined by the user, iterating over the zip codes in the order in which they enter the circle. In this way, both small and large circles are considered, all of which overlap with many other circles. The height of the cylinder represents the number of days, with the requirement that the last day is always included together with a variable number of preceding days, up to some maximum defined by the user. For example, we may consider all cylinders with a height of 1, 2, 3, 4, 5, 6, or 7 d. For each center and radius of the circular cylinder base, the method iterates over all possible temporal cylinder lengths. This means that we will evaluate cylinders that are geographically large and temporally short, forming a flat disk, those that are geographically small and temporally long, forming a pole, and every other combination in between.

What is new with the space–time permutation scan statistic is the probability model. Since we do not have population-at-risk data, the expected must be calculated using only the cases. Suppose we have daily case counts for zip-code areas, where *c_zd_* is the observed number of cases in zip-code area *z* during day *d*. The total number of observed cases (*C*) is



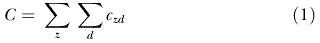



For each zip code and day, we calculate the expected number of cases μ*_zd_* conditioning on the observed marginals:



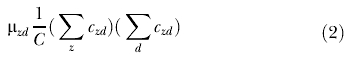



In words, this is the proportion of all cases that occurred in zip-code area *z* times the total number of cases during day *d*. The expected number of cases μ*_A_* in a particular cylinder *A* is the summation of these expectations over all the zip-code-days within that cylinder:



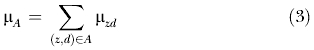



The underlying assumption when calculating these expected numbers is that the probability of a case being in zip-code area *z,* given that it was observed on day *d,* is the same for all days *d*.

Let *c_A_* be the observed number of cases in the cylinder. Conditioned on the marginals, and when there is no space–time interaction, *c_A_* is distributed according to the hypergeometric distribution with mean μ*_A_* and probability function



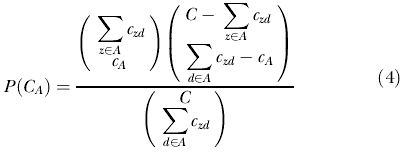



When both Σ*_z_*_ε*A*_ *c_zd_* and Σ*_d_*_ε*A*_ *c_zd_* are small compared to *C, c_A_* is approximately Poisson distributed with mean μ*_A_* [37]. Based on this approximation, we use the Poisson generalized likelihood ratio (GLR) as a measure of the evidence that cylinder *A* contains an outbreak:



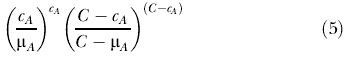



In words, this is the observed divided by the expected to the power of the observed inside the cylinder, multiplied by the observed divided by the expected to the power of the observed outside the cylinder. Among the many cylinders evaluated, the one with the maximum GLR constitutes the space–time cluster of cases that is least likely to be a chance occurrence and, hence, is the primary candidate for a true outbreak. One reason for using the Poisson approximation is that it is much easier to work with this distribution than the hypergeometric when adjusting for space by day-of-week interaction (see below), as the sum of Poisson distributions is still a Poisson distribution.

Since we are evaluating a huge number of outbreak locations, sizes, and time lengths, there is serious multiple testing that we need to adjust for. Since we do not have population-at-risk data, this cannot be done in any of the usual ways for scan statistics. Instead, it is done by creating a large number of random permutations of the spatial and temporal attributes of each case in the dataset. That is, we shuffle the dates/times and assign them to the original set of case locations, ensuring that both the spatial and temporal marginals are unchanged. After that, the most likely cluster is calculated for each simulated dataset in exactly the same way as for the real data. Statistical significance is evaluated using Monte Carlo hypothesis testing [38]. If, for example, the maximum GLR is calculated from 999 simulated datasets, and the maximum GLR for the real data is higher than the 50th highest, then that cluster is statistically significant at the 0.05 level. In general terms, the *p*-value is *p* = *R/*(*S* + 1) where *R* is the rank of the maximum GLR from the real dataset and *S* is the number of simulated datasets [38]. In addition to *p*-values, we also report null occurrence rates [8], such as once every 45 d or once every 23 mo. The null occurrence rate is the expected time between seeing an outbreak signal with an equal or higher GLR assuming that the null hypothesis is true. For daily analyses, it is defined as once every 1*/p* d. For example, under the null hypothesis we would at the 0.05 level on average expect one false alarm every 20 d for each syndrome under surveillance.

Because of the Monte Carlo hypothesis testing, the method is computer intensive. To facilitate the use of the methods by local, state, and federal health departments, the space–time permutation scan statistic has been implemented as a feature in the free and public domain SaTScan software [36].

### Implementation for New York City Syndromic Surveillance

Depending on the application, the method may be used with different parameter settings. For the syndromic surveillance analyses we set the upper limit on the geographical size of the outbreak to be a circle with a 5-km radius, and the maximum temporal length to be 7 d. This means that we are evaluating outbreaks with a circle radius size anywhere between 0 km (one zip code only) and 5 km, and a time length (cylinder height) of 1 to 7 d. The latter restriction is a reflection of the belief that the main purpose of this syndromic surveillance system is early disease outbreak detection, and if the outbreak has existed for over 1 wk, it is more likely to be picked up by reporting of specific disease diagnoses by clinicians or laboratories.

Another practical choice is the total number of days to include in the analysis. One option is to include all previous days for which data are available. We chose instead to analyze the last 30 d of data, adding one day and removing another for each daily analysis. We believe this time frame provides enough baseline beyond the 1- to 7-d scanning window to establish the usual pattern of visits while avoiding inclusion of data that may no longer be relevant to the current period.

To reduce the computational load, we limited the centers of the circular cylinder bases to be a collection of 446 zip-code area centroids and hospital locations in New York City and adjacent areas. This ensures, among other things, that each zip-code area may constitute an outbreak on its own.

The last parameter that we need to set is the number of Monte Carlo replications used for each analysis. For the daily prospective analyses we chose 999, which meant that the smallest *p*-value we could get was 0.001, so that the smallest null occurrence rate possible for a signal was once every 2.7 y. In our system, signals of that strength clearly merit investigation. For the historical evaluation, in order to obtain more precise null occurrence rates, we set the number of replications to 9,999.

### Adjusting for Space by Day-of-Week Interaction

The space–time permutation scan statistic automatically adjusts for any purely spatial and purely temporal variation. For many syndromic surveillance data sources, there is also natural space by day-of-week interaction in the data that is not due to a disease outbreak but to consumer behavior, store hours, etc. For example, if a particular pharmacy has an exceptionally high number of sales on Sundays because neighboring pharmacies are closed, we might get a signal for this pharmacy every Sunday unless we adjust for this space by day-of-week interaction. This can be done through a stratified random permutation procedure.

The first step is to stratify the data by day of week: Monday, Tuesday,…, Sunday. The space–time permutation randomization step is then done separately for each day of the week. For each zip code and day combination, the expected is then calculated using only data from that day of the week. For each cylinder, both the observed and expected number of cases is summed over all day-of-week strata, zip code, and day combinations within that cylinder. The same technique can be used to adjust for other types of space–time interaction. The underlying assumption when calculating these expected numbers is now that the probability of a case being in zip-code area *z,* given that it was observed on a Monday, is the same for all Mondays, etc.

All our analyses were adjusted for space by day-of-week interaction.

### Missing Data

Daily disease surveillance systems require rapid transmission of data, and it may not be possible to get complete data from each provider every single day. When we first tried the new method in New York City, a number of highly significant outbreak signals were generated that were artifacts of previously unrecognized missing or incomplete data from one or more hospitals. This is a good reflection on the method, since it should be able to detect abnormalities in the data no matter what their cause, but it also illustrates the importance of accounting for missing data in order to create an early detection system that is useful on a practical level.

Depending on the exact nature of the missing data, there are different ways to handle it. We used a combination of three different approaches. (1) If a hospital had missing data for all of the past 7 d (all possible days within the cylinder), we completely removed that hospital from the analysis, including all previous 23 d. (2) If a hospital had no missing data during the last 7 d, but one or more missing days during the previous 23 baseline days, then we completely removed the baseline days with some missing data, for all of the hospitals. (3) If a hospital had missing data for at least one but not all of the last 7 d, then we removed those missing days together with all previous days for the same hospital and the same day of week. That is, if Monday was missing during the last week, then we removed all Mondays for that hospital. This removal introduces artificial space by day-of-week interaction, so this approach only works if it is implemented in conjunction with the stratified adjustment for space by day-of-week interaction.

For some analyses, more than one of these approaches were used simultaneously. Note that, since the missing data depend on the hospital, the solution is to remove specific hospitals and days rather than zip codes and days, even when we are doing the zip-code-based residential analyses. If there are many hospitals with missing data, then the second approach could potentially remove all or almost all of the baseline days. To avoid this, one could sometimes go further back in time and add the same number of earlier days to compensate. Another option is to impute into the cells with missing data a Poisson random number of cases generated under the null hypothesis. Given the completeness of our data, neither of these methods were employed (94% of analyses were conducted with four or fewer baseline days removed).

## Results

### Evaluation Using Historical Data: Diarrhea Surveillance

We first tested the new method by mimicking daily prospective analyses of hospital emergency department data from 15 Nov 2001 to 14 Nov 2002, looking at diarrhea visits. Signals with *p ≤* 0*.*0027 are listed in Table 1 and depicted on the map in Figure 1. That is, we only list those signals with a null occurrence rate of once every year or less often.

**Figure 1:**
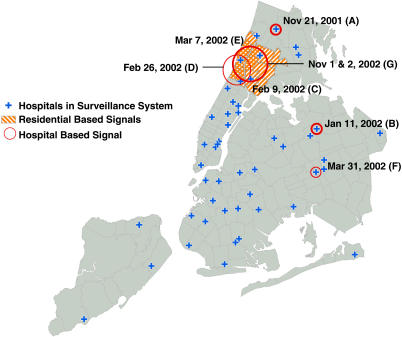
Locations and Dates of Detected Diarrhea Outbreak Signals, Using Historical Data from 15 November to 14 November 2002 The three stronger hospital-based signals are depicted with thicker lines/circles. The stronger residential-based signal was signal C. Note that all the zip-code areas in the residential signal E are also part of signal C.

**Table 1:**
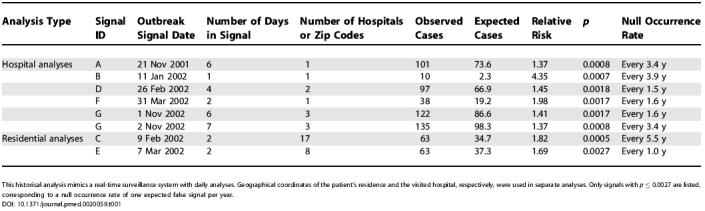
Analyses of Emergency Department Visits from 15 November 2001 to 14 November 2002 Due to Diarrhea

This historical analysis mimics a real-time surveillance system with daily analyses. Geographical coordinates of the patient's residence and the visited hospital, respectively, were used in separate analyses. Only signals with *p ≤* 0*.*0027 are listed, corresponding to a null occurrence rate of one expected false signal per year

For the residential zip-code analyses, there were two such signals. For the hospital analyses, there were six, two of which occurred in the same place on consecutive days. It is worth noting that at the false alarm rate chosen, none of the residential signals correspond to any of the hospital signals.

For the residential analysis, the strongest signal was on 9 February 2002, covering 17 zip-code areas in southern Bronx and northern Manhattan. This signal had 63 cases observed over 2 d when 34.7 were expected (relative risk = 1.82). With a null occurrence rate of once every 5.5 y, a spike in cases of this magnitude is unlikely to be due to random variation. The signal immediately preceded a sharp increase in citywide diarrheal visits from 10 February to 20 March (Figure 2). In both the localized 9 February cluster and the citywide outbreak, the increase was most notable among children less than 5 y of age. The weaker 26 February hospital signal and the 7 March residential signal that were centered in northern Manhattan occurred at the peak of this citywide outbreak. Laboratory investigation of the citywide increase in diarrheal activity indicated the rotavirus as the most likely causative agent.

**Figure 2:**
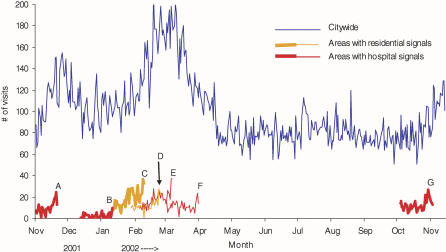
The Daily Temporal Pattern of Emergency Department Diarrhea Syndrome Visits in New York City, 1 November to 14 November 2002 For the citywide line (blue), daily counts are provided for the whole year. For each local area with a signal, daily counts are provided for the 1-mo period leading up to and including the day of the signal. The four stronger signals are depicted with thicker lines.

The two hospital signals on 1 November and 2 November 2002, were at the same three hospitals in southern Bronx and northern Manhattan, with null occurrence rates of 1.6 and 3.4 y, respectively. These signals immediately preceded another sharp increase in citywide diarrheal activity, this time among individuals of all ages (Figure 2). This citywide outbreak lasted approximately 6 wk and coincided with a number of institutional outbreaks in nursing homes and on cruise ships. Laboratory investigation of several of these outbreaks revealed the norovirus as the most likely causative agent. A similar citywide outbreak of norovirus in 2001 began shortly before the 21 November 2001 hospital signal in northern Bronx, which had a null occurrence rate of once every 3.4 y.

For the hospital analyses, the strongest signal was a 1-d cluster at a single hospital in Queens on 11 January 2002, with ten diarrhea cases when only 2.3 were expected, which one would only expect to happen once every 3.9 y. Being very local in both time and space, it is different from the previously described signals preceding citywide outbreaks. While examination of individual-level data revealed a predominance of infants under the age of two, this cluster could not be associated with any known outbreak, and retrospective investigation was not feasible.

As shown in Table 1, at the *p* = 0.0027 threshold there were six and two signals for the hospital and residential analyses, respectively, compared to one expected in each. Figure 3 shows the number of days on which the *p*-value of the most likely cluster was within a given range. Had the null hypothesis been true on all 365 d analyzed, the *p*-values would have been uniformly distributed between zero and one. The fact that in our data there were more days with low rather than high *p*-values is an indication that there may be additional true “outbreaks” that are indistinguishable from random noise. These could be very small disease outbreaks, for example, due to spoiled food eaten by only a few people, or they could be artifacts caused by, for example, changes in the hours of operation at an emergency department or coding differences between the emergency department triage nurses.

**Figure 3:**
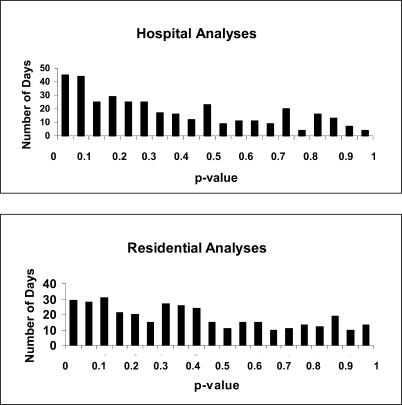
The Number of Days from 15 November 2001 to 14 November 2002 when the *p*-Value of the Most Likely Emergency Department Diarrhea Cluster Fell within the Interval Indicated for Both the Hospital (Top) and Residential (Bottom) Analyses

### Daily Prospective Surveillance

Since 1 November 2003, the space–time permutation scan statistic has been used daily in parallel with the population-at-risk-based space–time scan statistics [7] as part of the DOHMH Emergency Department surveillance system. For respiratory symptoms, fever/flu, and diarrhea, the results for the last week of November are listed in Tables 2 and 3. For diarrhea or respiratory symptoms there were no strong signals warranting an epidemiological investigation, and all had null occurrence rates of more often than once every month. This reflects a very typical week.

**Table 2:**
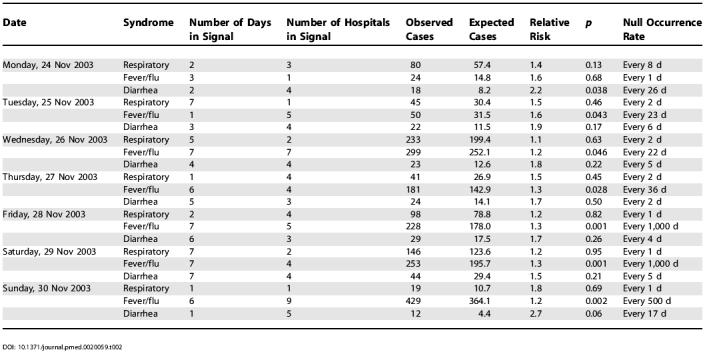
Real-Time Analyses of Emergency Department Visits Due to Diarrhea, Fever/Flu, and Respiratory Syndromes on Selected Days in November 2003, Using the Geographical Coordinates of the Hospital

**Table 3:**
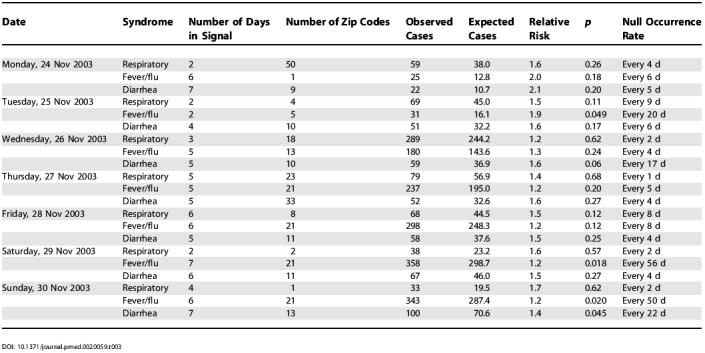
Real-Time Analyses of Emergency Department Visits Due to Diarrhea, Fever/Flu, and Respiratory Syndromes on Selected Days in November 2003, Using the Geographical Coordinates of the Patient's Residence

For fever/flu there was a strong 7-d hospital signal in southern Bronx and northern Manhattan on 28 November with a null occurrence rate of once every 2.7 y. On each of the following 2 d, there were again strong hospital signals in the same general area as well as residential zip-code signals of lesser magnitude. These signals started 12 d into a gradual citywide increase in fever/flu that continued to grow through the end of December, driven by an unusually early influenza season in New York City.

## Discussion

In this paper we have presented a new method for prospective infectious disease outbreak surveillance that uses only case data, handles missing data, and makes minimal assumptions about the spatiotemporal characteristics of an outbreak. When using historical emergency department chief complaint data to mimic a prospective surveillance system with daily analyses, we detected four highly unusual clusters of diarrhea cases, three of which heralded citywide gastrointestinal outbreaks due to rotavirus and norovirus. Three of four weaker signals also occurred immediately preceding or concurrent with these citywide outbreaks. If we assume that all of these clusters were associated with the citywide disease outbreaks, then the method generated at most two false alarms at a signal threshold where we would have expected one by chance alone.

For disease outbreak detection, the public-health community has historically relied on the watchful eyes of physicians and other health-care workers. However, the increasing availability of timely electronic surveillance data, both reportable diagnoses and pre-diagnostic syndromic indicators, raises the possibility of earlier outbreak detection and intervention if suitable analytic methods are found. While it is still unclear whether systematic health surveillance using syndromic or reportable disease data will be able to quickly detect a bioterrorism attack [39,40], the methods described here can also be applied to early detection of outbreaks of other, more common infectious diseases.

There are other alternative ways to calculate expected counts from a series of case data. One naive approach is to use the observed count 7 d ago in a zip-code area as the expected count for that same area today, and then apply the regular Poisson-based space–time scan statistic. When applied to the New York City diarrhea data described above, such an approach generated at least one “statistically significant” outbreak signal on each of the 365 d evaluated. The basic problem with this is that there is random variation in the observed counts that are used to calculate the expected, which is not accounted for in the Poisson-based scan statistic. If we based the expected on the average of multiple prior weeks of data, we would get less variability in the expected counts and fewer false signals, but the problem would still persist, and as the number of weeks increase beyond a few months other problems may gradually arise due to, for example, seasonal trends or population size changes.

Computing time depends on the size of the dataset and the analysis parameters chosen. With 999 replications, the hospital analyses with 38 data locations take 7 s to run on a 2.5-MHz Pentium 4 computer, while the residential analyses using 183 zip-code area locations take 11 s. The same numbers for 9,999 replications are 27 and 57 s, respectively.

There are a number of limitations with the proposed method. The method is highly sensitive to missing or incomplete data. Our first implementation of the method resulted in a number of false alarms, and highlights the need for systematic data quality checks and the analytic adjustments described above. When excellent population-at-risk data are available, we expect the Poisson-based space–time scan statistic that utilizes this extra information to perform better than the space–time permutation scan statistic. If, however, the population-at-risk data are of poor quality or nonexistent, which is often the case, then the space–time permutation scan statistic should be used.

Since the space–time permutation scan statistic adjusts for purely temporal clusters, it can only detect citywide outbreaks if they start locally, but not if they occur more or less simultaneously in the whole city. Hence, it does not replace purely temporal surveillance methods, but rather complements them.

Finally, it is important to note that the geographical boundary of the detected outbreak is not necessarily the same as the boundary of the true outbreak. Since we used circles as the base for the scanning cylinder, all detected outbreaks are approximately circular. Other shapes of the scanning window are also available [36], but it has been shown that circular scan statistics are also able to detect noncircular outbreak areas [41]. The less geographically compact the outbreak is, though, the less power (sensitivity) there is to detect it. For example, using circles we cannot expect to pick up an outbreak that is very long and narrow such as a one-block area on each side of Broadway, stretching from southern to northern Manhattan.

The emergency department data used in this study also have some limitations. In addition to the citywide outbreaks, there were several institutional gastrointestinal outbreaks reported to DOHMH during the historical 1-y period but not detected in emergency department data using the space–time permutation scan statistic. One reported outbreak involved school children that went to the emergency department of a nonparticipating hospital. Other outbreaks went undetected because medical care was not sought in emergency departments. Most people with diarrhea do not go to the hospital emergency department. Rather, they call or go to their primary care physician, they visit the pharmacy to buy over-the-counter medication, or they may have symptoms that are so mild that they do not seek medical care. Further studies are needed to evaluate the strengths and weaknesses of different data sources.

The geographic units of analysis used were residential zip code and hospital location. It may be hard to detect outbreaks that affect only a small part of a single zip code, especially if the background rate of the syndrome is fairly high. Where available, the exact coordinates of a patient's residence can be used to avoid problems introduced when aggregating data. Furthermore, some outbreaks may not be clustered by place of residence, as in the case of an exposure occurring at the place of work or in a subway. Using the location of the hospital rather than residence may provide higher power to detect workplace-related outbreaks, but the only way to fully address this issue may be to conduct workplace surveillance.

In spite of these limitations, we have presented a new method for the early detection of disease outbreaks and illustrated its practical use. The primary advantages of the method are that it is easy to use, it only requires case data, it automatically adjusts for naturally occurring purely spatial and purely temporal variation, it allows adjustment for space by day-of-week interaction, and it is capable of handling missing data.

While the method was developed and applied in the context of syndromic surveillance, it may also be used for the early detection of diagnosed disease outbreaks, or for detecting changes in the pattern of chronic diseases, when population census information is unavailable, unreliable, or not available at the fine geographical resolution needed. The ability to perform disease surveillance without population-at-risk data is especially important in developing countries, where these data may be hard to obtain. The space–time permutation scan statistic could also be used for similar early detection problems in other fields, such as criminology, ecology, engineering, social sciences, and veterinary sciences.

**Patient Summary**

**Background**

Detecting disease outbreaks early means that health officials are better able to fight and contain them. Electronic patient records that can be analyzed with statistical methods in computer programs should help with disease surveillance and make it possible to detect outbreaks early without raising too many false alarms.

**Why Was This Study Done?**

The researchers who did this study have developed and operated real-time disease surveillance systems. In any such system, there will always be more disease cases in some places and time periods than in others, for example, because there are more people living there, or because there are more people of a certain type living there, like older people or children, who are more prone to get sick. The researchers were trying to develop a method that can discover outbreaks without the need to know about the structure of the population under surveillance.

**What Did the Researchers Do?**

They modified an existing method to make it work without data on the structure of the population under surveillance. They also found a way to deal with incomplete data, when, for example, one hospital did not report any data for a particular day.

**What Did They Find?**

When they applied the method to emergency room data from New York City, they found that it performs well: it seems to be able to detect real outbreaks early and not result in many false alarms.

**What Are the Limitations of the Method?**

The method can detect only outbreaks that start locally, not those that occur more or less simultaneously in the whole surveillance area. For some outbreaks—for example, those caused by exposure to an infectious agent in the subway—patients will not necessarily live in the same neighborhood or go to the same emergency room. The method will not detect outbreaks with very few cases, such as one case of small pox or three cases of anthrax, such as the anthrax bioterrorism attacks in the fall of 2001. And the method only works for diseases with early symptoms severe enough that people go to the emergency room. Efficient disease surveillance will need the parallel use of different methods, each with their own strengths and weaknesses.

**What Next?**

The method was developed as part of the New York City Department of Health and Mental Hygiene surveillance initiatives and is now being used every day to analyze emergency department records from 38 hospitals in the city. To facilitate wider use, the method has been integrated into a more diverse software called SaTScan that is freely available.

**Where Can I Find Out More?**

The following websites provide additional information on this and other methods.

Details on SaTScan and software for downloading: http://www.satscan.org/

United States Centers of Disease Control and Prevention Web page on electronic disease surveillance: http://www.cdc.gov/od/hissb/act_int.htm

National Syndromic Surveillance Conference: http://www.syndromic.org/index.html

National Bioterrorism Syndromic Surveillance Demonstration Program: http://btsurveillance.org/

The Real-Time Outbreak and Disease Surveillance Open Source Project: http://openrods.sourceforge.net/

## Abbreviations

DOHMH: New York City Department of Health and Mental Hygiene
GLR: generalized likelihood ratio
